# Supplementary material for: Gephebase, a database of genotype–phenotype relationships for natural and domesticated variation in Eukaryotes
Source: Nucleic Acids Res. 2019 Sep 23;48(D1):D696–703. doi: 10.1093/nar/gkz796 (PMC6943045; doi:10.1093/nar/gkz796)
Supplement: gkz796_Supplemental_Files [file gkz796_supplemental_files.zip › Supplementary Legends.pdf]

Supplementary Legends:

Table S1. Csv file. Complete Gephebase dataset as of April 2019 (1771 entries).

Data S1. R script (R version 3.4 and R Studio version 1.2.1335) used to create Fig. 4.
